# Over-expression of long non-coding RNA insulin-like growth factor 2-antisense suppressed hepatocellular carcinoma cell proliferation and metastasis by regulating the microRNA-520h/cyclin-dependent kinase inhibitor 1A signaling pathway

**DOI:** 10.1080/21655979.2021.1975016

**Published:** 2021-09-13

**Authors:** Zhen Huang, Guofeng Su, Xiaoxia Bi, Libo Zhang, Zhuohui Xu, Ge Wang

**Affiliations:** a Department of Interventional Radiology, Huizhou First Hospital, Guangdong, China; b Department of Medical Oncology, Huizhou First Hospital, Guangdong, China

**Keywords:** Hepatocellular carcinoma, insulin-like growth factor 2-antisense, microRNA-520h, cyclin-dependent kinase inhibitor 1A

## Abstract

Primary liver cancer is the sixth most common cancer and the third leading cause of malignancy-related death worldwide in 2020, with 75–85% of hepatocellular carcinoma (HCC). Evidences have verified that long noncoding RNAs (lncRNAs) play key roles in HCC onset and development. However, the function and mechanism of lncRNA insulin-like growth factor 2-antisense (IGF2-AS) in HCC remain unclear. Herein, IGF2-AS expression profile in HCC patients was first investigated based on The Cancer Genome Atlas (TCGA) database and local HCC patients, followed by prognostic value evaluation using Kaplan–Meier method; then, the bioinformatics analysis, dual-luciferase reporter assay, Spearman correlation assay, function gain, and loss with rescue experiments were applied to investigate the biological function and the involved molecular mechanisms of IGF2-AS in HCC oncogenesis and development. Our results showed that IGF2-AS expression was significantly down-regulated in HCC cells and tissues; lower IGF2-AS expression was significantly associated with poor prognosis of HCC patients; IGF2-AS over-expression inhibited the viability, colony formation, invasion, and migration, while promoted apoptosis *in vitro*, and inhibited HCC xenograft growth *in vivo*; IGF2-AS sponged microRNA-520h (miR-520h) to up-regulate IGF2-AS expression, and miR-520h over-expression or cyclin-dependent kinase inhibitor 1A (CDKN1A) silencing reversed IGF2-AS reduced aggressive behaviors of HCC cells. In conclusion, IGF2-AS is a tumor-suppressor in HCC, and lower IGF2-AS expression is associated with poor prognosis of HCC patients; IGF2-AS inhibits HCC oncogenesis and development by IGF2-AS/miR-520h/CDKN1A pathway. Therefore, IGF2-AS may serve as a new biomarker for HCC management.

## Background

Primary liver cancer is the sixth most commonly diagnosed malignancy and the third leading cause of malignancy-related death worldwide in 2020, with about 906,000 new patients and 830,000 deaths. Hepatocellular carcinoma (HCC) accounts for 75–85% of primary liver cancer [1]. Owing to the insidiousness of HCC onset and lack of effective treatment methods, the prognosis of HCC is extremely poor, and the 5-year average survival rate is less than 10% [2]. It is necessary to identify more effective molecular targets or combination therapeutics for the diagnosis and treatment of HCC.

**Figure 1.**
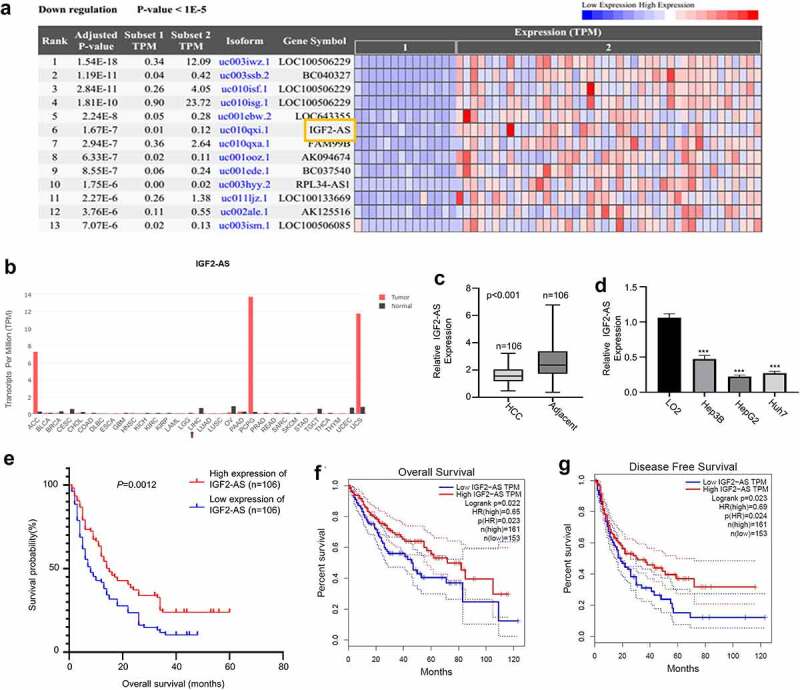
IGF2-AS expression is down-regulated significantly in HCC cells and tissues, and related with prognosis of HCC patients. IGF2-AS expression was down-regulated in cancer (t) than in normal (n) tissues of HCC patients investigated by bioinformatics assay (a) based on TCGA datasets; (b) analyzed using GEPIA online tool; (c) IGF2-AS expression was dramatically down-regulated in HCC than in adjacent tissues from 106 local HCC patients detected by qRT-PCR assay. (d) IGF2-AS expression was dramatically down-regulated in human HCC (Hep3B, HepG2 and Huh7) than in human normal liver (LO2) cells detected by qRT-PCR, and HepG2 and Huh7 cells had the lowest IGF2-AS expression level. (e) Kaplan-Meier curve showed that low IGF2-AS expression was associated with poor prognosis in overall survival of HCC patients (n = 80). Low IGF2-AS expression was significantly associated with decreased (f) overall survival and (g) disease free survival of HCC patients from the TCGA database. *p < 0.05, **p < 0.01, ***p < 0.01

Increasing studies have verified that long noncoding RNAs (lncRNAs) play key roles during the development of multiple human cancers [3–5]. Studies have reported that IGF2-AS is a biomarker for cancer diagnosis and prognosis, including as a tumor suppressor in breast cancer [6], a promotor of glycolysis and apoptosis of gastric cancer cells via targeting miR-195/CREB1 axis [7], a potential target for colorectal cancer (CRC) treatment [8], as well as the prognosis and metastasis of gastric adenocarcinoma via serving as a ceRNA of miR-503 to regulate SHOX2 9. We identified that IGF2-AS was remarkably down-regulated in tumor tissues by analyzing the differentially expressed lncRNAs in HCC based on the RNA-seq dataset in TCGA database (14 tumor and 42 adjacent normal samples) (Figure 1a). However, the roles of IGF2-AS in HCC still remain unknown. Therefore, we decided to investigate its biological function and mechanism of IGF2-AS in HCC.

Accumulating evidences suggest that lncRNAs mainly serve as competing endogenous RNAs (ceRNAs), and compete with microRNAs (miRNAs) to regulate mRNAs [9,10]. However, whether IGF2-AS regulates HCC oncogenesis and metastasis via ceRNA mechanism remains to be explored, herein, we first carried out the bioinformatics analysis to predict the potential miRNAs could specifically bind to IGF2-AS-3ʹUTR sequence, and the downstream target mRNA. After bioinformatics analysis based on Starbase 2.0 database (http://starbase.sysu.edu.cn/), miR-520h was identified to have the potential complementary binding sites with the IGF2-AS-3ʹUTR sequence (Figure 3a); furthermore, we identified that miR-520h expression in liver cancer was significantly higher than that of adjacent normal tissues in TCGA database analyzed with the Starbase 2.0 on line tool (http://starbase.sysu.edu.cn/) (Figure 3b). Moreover, miR-520h has been predicted to have potential binding sites with cyclin-dependent kinase inhibitor 1A (CDKN1A)-3ʹUTR sequence based on Starbase 2.0 online website (Figure 4a).

Based on the aforementioned information, we hypothesized that over-expression of lncRNA IGF2-AS suppressed HCC development by targeting miR-520h/CDKN1A signaling pathway. Therefore, in this study, we aimed to investigate the roles and the ceRNA mechanisms of IGF2-AS in HCC carcinogenesis and development.

### Patients and methods

#### Patient specimens

Cancer and self-matched adjacent tissues were collected from HCC patients who received surgery treatment in our hospital between January 2019 to August 2020.

#### Reagents

DMEM/high glucose medium and 1% penicillin–streptomycin (Hyclone, Logan, UT, USA); antibodies against CDKN1A (#2947 at 1:2000 for Western Blotting, at 1:50 for Immunohistochemistry), Ki-67 (#12,202 at 1:200 for Immunohistochemistry) and GAPDH (#5174 at 1:1000 for Western Blotting) (Cell Signaling Technology, Danvers, MA, USA); Dual-Luciferase Reporter Assay System (Promega, Madison, WI, USA); matrigel (BD, New Jersey, USA); CCK-8 test kit (Dojindo Corp, Kyushu, Japan); PrimeScrip™ RT Master Mix, SYBR Premix Ex Taq I and PrimeScript miRNA cDNA Synthesis Kit (Fisher BioReagents®, USA); lipofectamine 2000, pmirGLO vector, fetal bovine serum (FBS), pcDNA3.1 vector and Trizol reagent (Invitrogen, USA).

#### Ethics and informed consents

This study got permission from Ethical Committee on Scientific Research of Huizhou First People’s Hospital. All included HCC patients provided written informed consents.

#### Cells and cell culture

A human normal liver (LO2) and three human HCC (Hep3B, HepG2 and Huh7) cell lines were bought from American Type Culture Collection (Manassas, VA, USA) and maintained in Roswell Park Memorial Institute (RPMI) 1640 medium with 10% FBS and 1% penicillin–streptomycin in a humidified atmosphere at 37°C containing 5% CO_2_[11].

#### Oligonucleotides, vectors and transfection

Guangzhou Ribobio Co., Ltd. (Guangzhou, China) provided small interfering RNA (siRNA) for negative control (NC) and siRNA against CDKN1A. The negative control of miR-520h mimics (miR-NC), miR-520h mimics, negative control of miR-520h inhibitor (miR-NC), and miR-520h inhibitor were designed and synthesized by Genechem (Shanghai, China).

Full-length IGF2-AS cDNA was cloned into pcDNA3.1 vector. Empty (NC) and IGF2-AS containing pcDNA3.1 vector (IGF2-AS) were transfected into HepG2 and Huh7 cells, respectively, using Lipofectamine 2000, and 2 mg/ml of G418 was used to screen stably transfected cells.

The pLKO.1 vector was served as negative control (NC) for CDKN1A silence. IGF2-AS or CDKN1A 3′-UTR sequence covering mutant (mut) or wild-type (WT) binding sites of miR-520h were respectively cloned into dual-luciferase reporter vector (pmirGLO), Lipofectamine 2000 was used to transfect NC, IGF2-AS siRNA or miR-520h mimics [11].

#### Dual-luciferase reporter assay

Based on bioinformatics assay, miR-520h has the potential binding site with CDKN1A 3′-UTR. pmirGLO vector was applied to confirm direct binding between miR-520h with 3′-UTR of IGF2-AS (or 3′-UTR of CDKN1A). Constructs of mutant (mut) reporter (pmirGLO/IGF2-AS 3′-UTR-mut or pmirGLO/CDKN1A 3′-UTR-mut) and wild-type (WT) reporter (pmirGLO/IGF2-AS3′-UTR or pmirGLO/CDKN1A 3′-UTR) were co-transfected with miR-520h negative control (NC) or miR-520h mimics in HepG2 and Huh7 cells. Firefly luciferase activity was determined with microplate reader, and the final luciferase activity of report gene was calculated after normalizing to Renilla luciferase activity [11].

#### Total RNA extraction and qRT-PCR assay

Total RNA was extracted with Trizol reagent. The cDNA was reverse transcribed with PrimeScrip™ RT Master Mix. Reverse transcription for miRNAs was performed with PrimeScript miRNA cDNA Synthesis Kit. Relative RNA expression was calculated by 2^−ΔΔ*C*t^ method [12,13].

#### Cell viability assessment

Cell viability was evaluated by CCK-8 assay as described in the CCK-8 Kit. Briefly, cells to be tested were plated at an initial density of 1 × 10^4^ cells/well per 100 μL of cell culture medium in 96-well plates; 10 μL of CCK-8 reagent was added into 100 μL of cell culture medium. The optical density (OD) values were measured at 450 nm using a microplate reader [12,14].

#### Wound healing migration analysis

A wound was scratched at the middle of the 6-well plate with 100% confluent monolayer cells using a 10-µl pipette tip. After being washed with sterile PBS to remove the detached cells, the remaining cells were then cultured in fresh serum-free medium for 24 h at 37°C. Images for cell migration at 0 and 24 h were obtained under an inverted light microscope (Olympus, Japan). The relative migration distance was quantified by measuring the migrated cell surface width during 24 h and calculated as: Relative migration distance = the migrated cell surface width during 24 h of test cells/the migrated cell surface width during 24 h of control cells [15].

#### Colony formation evaluation

Cells (1000/well) were plated into 6-well plates and cultured for 10 days. The colonies were fixed with 4% paraformaldehyde for 10 min and staining with 0.5% crystal violet for 5 min, and then counted after photographing [11,12].

#### Transwell invasion assay

Matrigel coated 24-well transwell chambers (Corning Costar, USA) was used to estimate the invasion ability. In brief, 750 μL of medium containing 10% FBS was used as a chemoattractant and loaded in lower chamber, 3 × 10^5^ cells in 200 μl of serum-free medium was loaded in upper insert and cultured for 48 h. Invaded cells on lower surface of upper insert were fixed with 4% paraformaldehyde and stained with 0.1% crystal violet [12].

#### Western blot assay

Total proteins were extracted with RIPA Cell Lysis Buffer on ice following the manufacturer’s protocol. The supernatant (total protein) was collected after centrifuging at 4°C and 13,000 g for 15 min. The protein concentration was measured using the BCA method following the manufacturer’s protocol. Then, equal amount proteins (15 μg/sample) were loaded and separated on 10% SDS-PAGE, transferred to PVDF membrane, blocked with 5% fat free milk, blotted with primary antibody (antibody against CDKN1A at 1:2000, antibody against Ki-67 at 1:200 or antibody against GAPDH at 1:1000) at 4°C overnight and secondary antibody at room temperature for 1 h, respectively, developed with SuperSignal Chemiluminescent HRP Substrate, and photographed with software Image Lab version in ChemiDoc MP Imaging System (Bio-Rad, USA) [11,12,14].

#### Animal experiments

The animal studies were approved by the Biomedical Ethics Committee of Huizhou First People’s Hospital. Ten six-week-old female BALB/c nude mice were bought from Beijing HFK Bioscience Co. Ltd. (Beijing, China), separated into two groups (*n* = 5) and maintained under standard conditions (12 h dark/light cycle, 50–80% humidity, and 15–27°C pathogen-free) in Huizhou First People’s Hospital. To establish the xenograft model in nude mice, HepG2 cells over-expressed IGF2-AS or NC were re-suspended in 200 μl PBS, respectively, and injected subcutaneously (1 × 10^6^ cells per mouse) into flank of each mouse. The minimum (W) and maximum (L) length of tumors were measured with a vernier caliper every week to calculate the tumor volume with the formula of ½LW [2]. The mice were sacrificed by cervical dislocation after 5 weeks; the tumor tissues were isolated for immunohistochemical assay [14].

#### Immunohistochemical assay

Tumor tissues were fixed in 10% formalin and embedded in paraffin, sliced into 4 μm sections. Then the paraffin-embedded sections were deparaffinized with xylene, followed by rehydrating with alcohol, blocking endogenous peroxidase with 3% H_2_O_2_, antigen retrieval by microwave heating, blocking nonspecific antigen in 5% BSA, incubating with primary antibody against Ki-67 or CDKN1A and then biotinylated secondary. The peroxidase reaction was visualized using 3,3′- diaminobenzidine tetrahydrochloride (DAB) and counterstained using hematoxylin. Images were photographed under microscope. The total and positive cell numbers were counted in five randomly selected fields of each section under a microscope at 400× magnification to quantitate the staining intensity. The positive cell percentages from five random fields were used for statistics analysis [14].

### Statistical analysis

Statistics were analyzed with SPSS 20.0 (IBM, Chicago, IL, USA) with Student’s *t*-test for two groups and one-way ANOVA for multiple group comparison. Kaplan–Meier method was used for overall survival rate comparison. Association between different genes was analyzed with Spearman’s correlation coefficient. *p* < 0.05 indicates statistical significance [14].

## Results

This study was aimed to reveal the biological role and the ceRNA mechanism of lncRNA IGF2-AS in regulating HCC carcinogenesis and development. After a comprehensive literature review and bioinformatics analysis, we postulated that over-expression of lncRNA IGF2-AS suppressed HCC development by targeting miR-520h/CDKN1A signaling pathway. IGF2-AS expression profile in HCC patients was investigated based on both the Cancer Genome Atlas (TCGA) database and the local HCC patients by qRT-PCR; prognostic value of IGF2-AS expression was evaluated using Kaplan–Meier method; the biological function of IGF2-AS was determined by viability, colony formation, invasion, migration and apoptosis of HCC cells *in vitro*, and HCC xenograft growth *in vivo* with or without IGF2-AS over-expression; the specific molecular mechanisms of IGF2-AS in HCC carcinogenesis and development were investigated by bioinformatics analysis, dual-luciferase reporter assay, Spearman correlation assay, function gain and loss with rescue experiments.

### Significantly down-regulated IGF2-AS expression is identified in HCC patient tissues and cells, and associated with poor prognostic outcome of HCC patients

For investigating the biological function of IGF2-AS in HCC oncogenesis and progression, we first extracted IGF2-AS expression profile in tissues of HCC patients from TCGA (Figure 1a) and GEPIA (Figure 1b) databases, which revealed a significantly down-regulated IGF2-AS expression in tumors tissues than in normal tissues of HCC patients. Moreover, we collected HCC and self-matched adjacent tissues from 106 local HCC patients and performed qRT-PCR to confirm the above findings. The detailed clinicopathologic features of included HCC patients have been shown in Table 1. IGF2-AS expression was also found to be significantly down-regulated in HCC than in adjacent tissues (Figure 1c). Down-regulated IGF2-AS expression was also confirmed in HCC cells, which showed that IGF2-AS expression was significantly lower in Hep3B, HepG2, and Huh7 cells versus LO2 cells, and IGF2-AS expression was lowest in HepG2 and Huh7 cells (Figure 1d). The median expression value of IGF2-AS in HCC tissues was used as cutoff value, then the 106 local HCC patients were divided into IGF2-AS high expression (*n* = 53) and low expression (*n* = 53) groups. Kaplan–Meier curves of IGF2-AS high or low expression HCC patients were then established, which identified a poor prognostic outcome of HCC patients with low IGF2-AS expression in overall survival (Figure 1e). Furthermore, the association between IGF2-AS expression and the prognosis of HCC patients were analyzed using TCGA database to confirm the prognostic value of IGF2-AS expression. The data showed that decreased IGF2-AS expression was significantly associated with decreased overall survival (Figure 1e) and disease-free survival (figure 1f) of HCC patients from the TCGA database. Overall, these findings indicated that IGF2-AS served as a tumor suppressor in HCC. Moreover, lower IGF2-AS expression was found to be significantly associated with poor prognosis of HCC patients.

**Table 1.** Correlations of IGF2-AS expression with clinicopathologic features of hepatocellular carcinoma

Patients	High IGF2-AS (*n* = 53)	Low IGF2-AS (*n* = 53)	*P*-value
**Age (years)**	0.5589		
≥55	26	23	
<55	27	30	
**Gender**	0.1185		
Female	25	33	
Male	28	20	
**Vascular invasion**	0.0063**		
Yes	22	36	
No	31	17	
**Race**	0.7501		
Yellow	48	47	
Others	5	6	
**Lymph node metastasis**	0.0192*		
No	30	18	
Yes	23	35	
**TNM stage**	0.0091**		
I	22	8	
II	11	12	
III	18	10	
IV	12	23	
**Hepatitis B history**	0.1662		
Yes	28	35	
No	25	18	
**Liver cirrhosis history**	0.3139		
Yes	31	36	
No	22	17	
**Tumor size**	0.0296*		
≤5 cm	37	26	
>5 cm	16	27	
**Tumor number**	0.0115*		
Single	34	21	
Multiple	19	32	
**AFP level (ng/L)**	0.0113*		
≤400	35	22	
>400	18	31	

### Over-expression of IGF2-AS inhibits viability and metastasis, while promotes apoptosis of HCC cells

**Figure 2.**
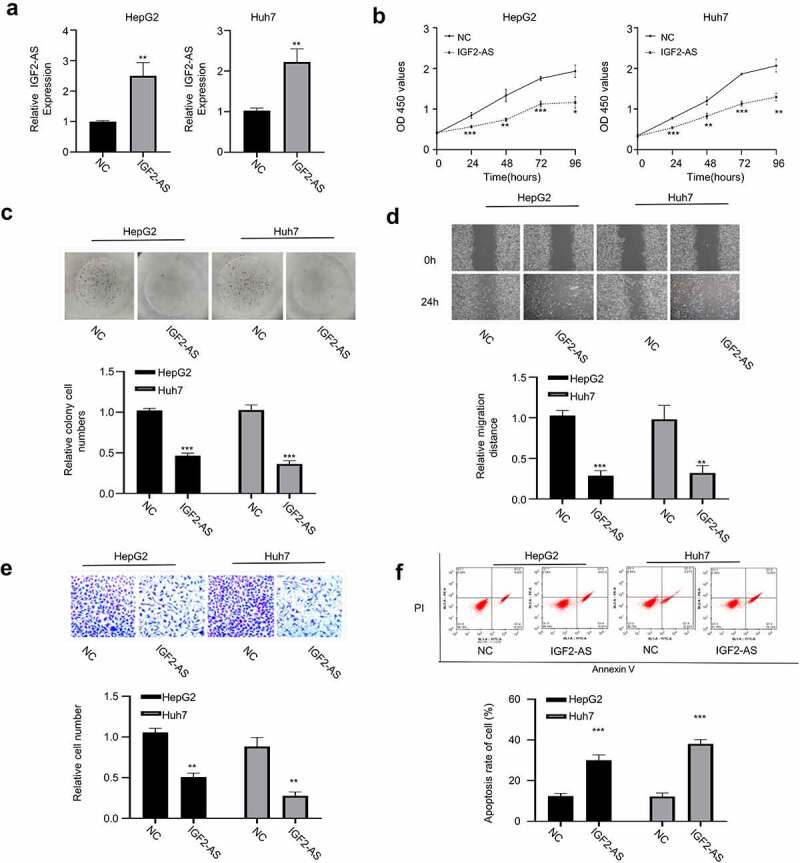
IGF2-AS over-expression inhibits proliferation and migration, while promotes apoptosis of HCC cells. IGF2-AS was over-expressed successfully in HepG2 and Huh7 cells (a), then viability (b), colony formation(c), migration(d), invasion(e) and cell apoptosis (f) were investigated and compared between cells with (IGF2-AS) or without (NC) IGF2-AS over-expression. **p < 0.01

To determine function of IGF2-AS in HCC cells, HepG2, and Huh7 cells were transfected respectively with pcDNA3.1 vector containing IGF2-AS sequence to over-express IGF2-AS, and empty pcDNA3.1 vector was used as the negative control (NC). The qRT-PCR analysis showed that IGF2-AS was successfully over-expressed in both HepG2 and Huh7 cells after transfection of IGF2-AS containing pcDNA3.1 vector versus the NC (Figure 2a). After IGF2-AS over-expression, the aggressive HCC phenotypes were detected in both HepG2 and Huh7 cells and compared. Viabilities (0–96 h) of HepG2 and Huh7 cells were detected with CCK-8 assay, which showed that IGF2-AS over-expression time dependently inhibited cell viability compared with the NC (Figure 2b). Cell colony formation ability was significantly decreased with IGF2-AS over-expression in HepG2 and Huh7 cells versus the NC (Figure 2c). Cell migration ability was determined by wound-healing assay, which showed that IGF2-AS over-expression in HepG2 and Huh7 cells significantly suppressed cell migration ability (Figure 2d). Cell invasion ability was determined by transwell assay, which showed that IGF2-AS over-expression in HepG2 and Huh7 cells significantly suppressed cell invasion ability (Figure 2e).Cell apoptosis percentage was determined by flow cytometric assay, which showed a significant increased apoptosis after IGF2-AS over-expression versus the NC in both HepG2 and Huh7 cells (figure 2f).

### LncRNA IGF2-AS acts as a sponge for miR-520h

**Figure 3.**
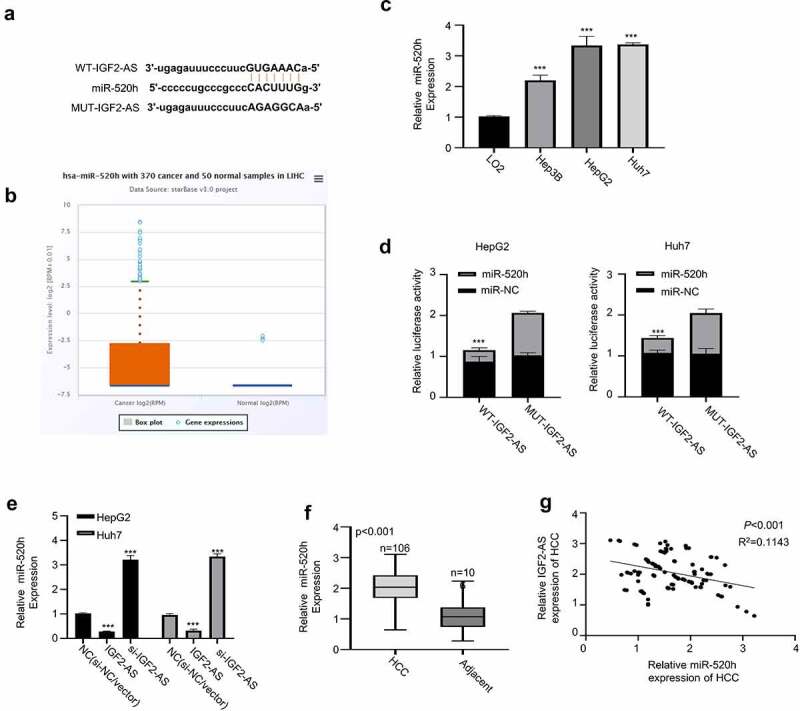
IGF2-AS acts as a sponge for miR-520h. (a) Illustration of prospective miR-520h binding sites in IGF2-AS-3ʹUTR predicted by Starbase 2.0 database and the mutation sites used for specific binding assay (http://starbase.sysu.edu.cn/). (b) miR-520h expression in liver cancer was significantly higher than that of adjacent normal tissues in TCGA database analyzed with the Starbase 2.0 on line tool (http://starbase.sysu.edu.cn/) (*p* = 0.0017). (c) miR-520h expression in HepG2 and Huh7 cells was significantly higher than that in LO2 cells detected by qRT-PCR. (d) Dual-luciferase reporter assay in HepG2 and Huh7 cells containing wild type (IGF2-AS) or mutated (MUT) reporter of IGF2-AS in the presence of miR-520h mimics or NC. (e) IGF2-AS expression in HepG2 and Huh7 cells after miR-520h was silenced with miR-520h inhibitor or over-expressed with miR-520h mimics. (f) miR-520h expression was dramatically up-regulated in HCC than in adjacent tissues from 106 HCC patients (same specimens as in Figure 1c). (F) Spearman’s coefficient assay showed a significantly negative association between IGF2-AS and miR-520h expressions in 106 HCC tissues (same specimens as in Figure 1c). **p < 0.01

Since lncRNAs have been reported to serve as ceRNAs in regulating biological functions by sponging miRNAs [16,17]. Therefore, we hypothesized that IGF2-AS may act as a ceRNA in HCC development. To prove this hypothesis, bioinformatics analysis was first accomplished to predict the prospective binding sites of miR-520h in IGF2-AS-3ʹUTR sequence based on Starbase 2.0 database (http://starbase.sysu.edu.cn/). As shown in Figure 3a, miR-520h was predicted to have potential binding sites in 3′-UTR of IGF2-AS. The expression of miR-520h in liver cancer was investigated to further identify the involved ceRNA, which revealed that miR-520h expression in liver cancer was significantly higher than that of adjacent normal tissues in TCGA database analyzed with the Starbase 2.0 on line tool (http://starbase.sysu.edu.cn/) (Figure 3b); meanwhile, miR-520h expression was identified to be significantly up-regulated in Huh7 and HepG2 cells than in LO2 cells analyzed by qRT-PCR (Figure 3c). Moreover, dual-luciferase reporter activity was measured to confirm binding ability between IGF2-AS 3′-UTR and miR-520h by sub-cloning IGF2-AS 3′-UTR with mutated or WT reporter gene into pmirGLO, the dual-luciferase reporter vector. The results showed that luciferase activities of pmirGLO-IGF2-AS 3′-UTR-WT reporter gene in both HepG2 and Huh7 cells were decreased significantly in the presence of miR-520h mimics, which were not changed after predicted miR-520h binding sites in IGF2-AS 3′-UTR were mutated (Figure 3d). The function of miR-520h in HCC was then explored, and IGF2-AS expression was confirmed to be negatively regulated by miR-520h expression. As we can see in Figure 3e, qRT-PCR assay showed that IGF2-AS expression in HepG2 and Huh7 cells was decreased in the presence of miR-520h mimics, while increased in the presence of miR-520h inhibitor. Moreover, miR-520h expression in HCC tissues was found to be significantly up-regulated (figure 3f) and negatively correlated to IGF2-AS expression in the HCC tissues from included 106 local HCC patients by Spearman’s coefficient assay (Figure 3g), indicating that miR-520h acted as an oncogenenic factor in HCC. Overall, these findings indicated that IGF2-AS was a ceRNA of miR-520h in HCC.

### IGF2-AS sponges miR-520h to up-regulate CDKN1A expression in HCC cells

**Figure 4.**
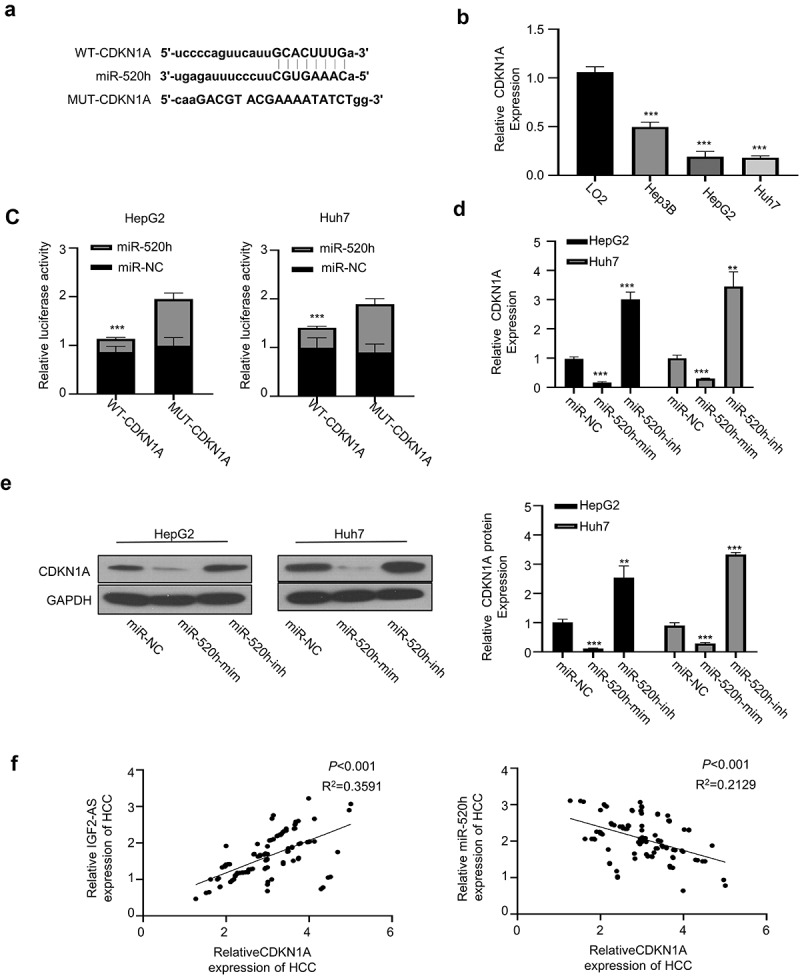
IGF2-AS elevates CDKN1A expression by sponging miR-520h. (a) Diagram of prospective miR-520h binding sites in CDKN1A-3ʹ-UTR predicted with miRTarBase (http://mirtarbase.mbc.nctu.edu.tw/php/index.php). (b) CDKN1A mRNA expression was significantly down-regulated in HCC (HepG2 and Huh7) than in normal liver (LO2) cells determined by qRT-PCR. (c) dual-luciferase reporter assay in HepG2 and Huh7 cells containing wild type (CDKN1A) or mutated (MUT) reporter of CDKN1A-3ʹ-UTR with miR-520h mimics or negative control (miR-NC). (d) CDKN1A mRNA expression in HepG2 and Huh7 cells after miR-520h was silenced with miR-520h inhibitor or over-expressed with miR-520h mimics. (e) CDKN1A protein expression in HepG2 and Huh7 cells after miR-520h was silenced with miR-520h inhibitor or over-expressed with miR-520h mimics. (f) Spearman’s correlation assay of positive correlations between CDKN1A with IGF2-AS expressions and negative correlations between CDKN1A with miR-520h expressions in 106 HCC tissues (same specimens used in Figure 1C). **p < 0.01

We found that CDKN1A 3′-UTR harbored the potential binding sites of miR-1303 by analyzing the miRTarBase (http://mirtarbase.mbc.nctu.edu.tw/php/index.php) (Figure 4a). Therefore, we hypothesized that miR-520h may directly bind to and regulate CDKN1A.

To further verify the direct binding between miR-520h with CDKN1A 3′-UTR, we first compared the CDKN1A expression between HCC (Hep3B, HepG2, and Huh7) and normal liver (LO2) cells, which showed that CDKN1A mRNA expression was significantly down-regulated in HCC cells than in normal liver cells (Figure 4b). The wild-type (WT) or mutated CDKN1A 3′-UTR reporter gene was then sub-cloned into pmirGLO plasmid for luciferase activity assay, which showed that luciferase activity of WT CDKN1A 3′-UTR reporter gene in both HepG2 and Huh7 cells was significantly inhibited in the presence of miR-520h mimics versus the miR-520h NC, while the luciferase activities did not change when the predicted binding sites of miR-520h in CDKN1A 3′-UTR were mutated (Figure 4c), suggesting that CDKN1A is the target of miR-520h. This direct binding was verified by checking mRNA (Figure 4d) and protein (Figure 4e) expressions of CDKN1A in HepG2 and Huh7 cells in the presence of miR-520h mimics or miR-520h inhibitor, which showed that CDKN1A mRNA expression was significantly inhibited by miR-520h mimics, while promoted by miR-520h inhibitor versus the NC. Moreover, Spearman’s correlation assay showed that CDKN1A expression in cancerous tissues from 106 local HCC patients (same specimens as in Figure 1c) was positively correlated with IGF2-AS expression, and negatively correlated with miR-520h expression (figure 4f). These findings indicated that CDKN1A was up-regulated after miR-520h was sponged by IGF2-AS in HCC cells.

### CDKN1A silencing reverses IGF2-AS-attenuated aggressive phenotypes of HCC

**Figure 5.**
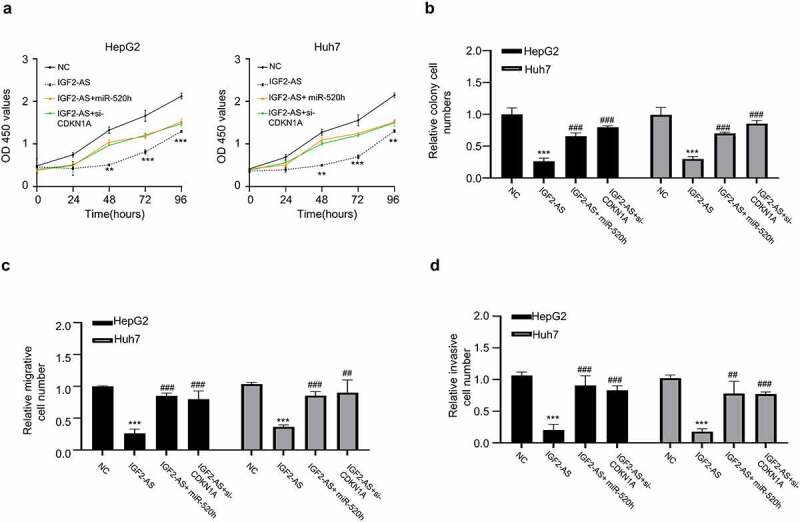
MiR-520h over-expression or CDKN1A silencing reverses IGF2-AS reduced aggressive behaviors of HCC cells. (a) viability, (b) colony formation, (c) migration, and (d) invasion abilities in HepG2 and Huh7 cells after IGF2-AS over-expression (IGF2-AS) with or without miR-520h over-expression by miR-520h mimics (miR-520h) or CDKN1A silencing by si-CDKN1A. **p < 0.01

To further study the function and mechanism of IGF2-AS/miR-520h/CDKN1A axis in HCC development, we performed the rescue experiments followed by analyzing the viability, colony formation, migration and invasion abilities in HepG2 and Huh7 cells. The data indicated that IGF2-AS over-expression significantly suppressed OD values of HepG2 and Huh7 cells (0–96 h), and the attenuated OD values were partly reversed in the presence of miR-520h mimics or si-CDKN1A (Figure 5a); the colony formation abilities of HepG2 and Huh7 cells were also significantly decreased with IGF2-AS over-expression, which was partly rescued when miR-520h mimics or si-CDKN1A was co-transfected (Figure 5b); transwell assay showed that cell migration abilities of HepG2 and Huh7 cells were also significantly decreased with IGF2-AS over-expression, which was partly rescued when miR-520h mimics or si-CDKN1A was co-transfected (Figure 5c); transwell assays showed that invasion abilities of HepG2 and Huh7 cells were inhibited after IGF2-AS over-expression, which also significantly reversed when miR-520h mimics or si-CDKN1A was co-transfected (Figure 5d). Therefore, over-expressed miR-520h or inhibited CDKN1A reversed the preventative effect of IGF2-AS in HCC development.

**Figure 6.**
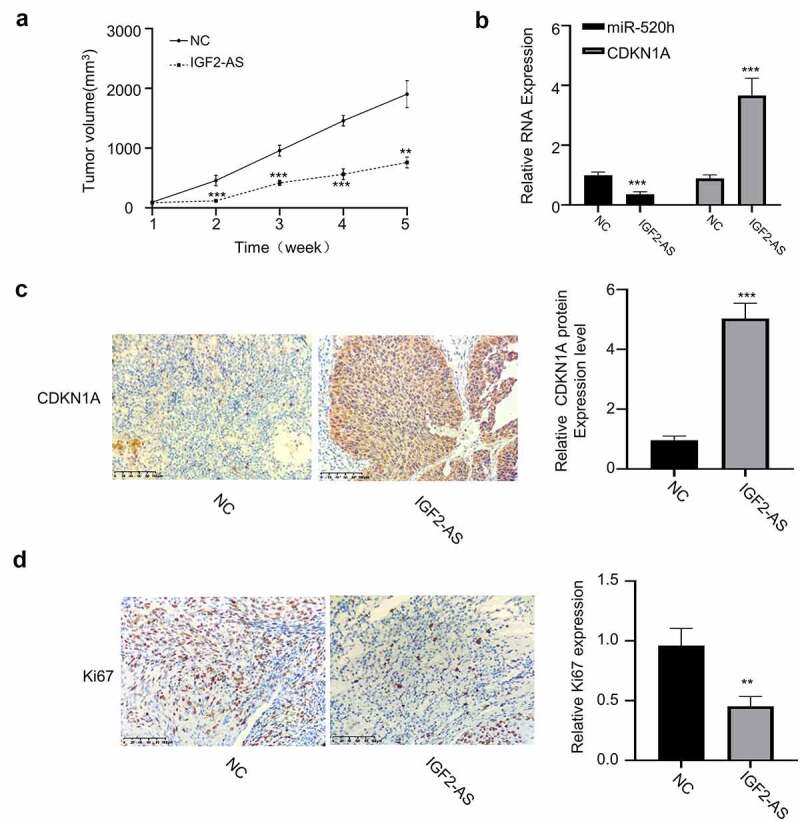
IGF2-AS over-expression in HepG2 cells inhibits xenograft tumor growth by regulating miR-520h/CDKN1A axis *in vivo*. (a) Tumor volume (*n* = 5 each). (b) miR-520h and CDKN1A expressions by qRT-PCR assay. (c) CDKN1A and (d) Ki-67 expressions by immunochemistry. **p < 0.01, ***p < 0.001,^##^*p* < 0.01, ^###^*p* < 0.001

Furthermore, xenograft models, generated by subcutaneous injection of IGF2-AS stably over-expressed HepG2 cells, were then applied to investigate biological function of IGF2-AS/miR-520h/CDKN1A axis *in vivo*. Same as the results *in vitro*, IGF2-AS over-expression considerably inhibited tumor volume versus those in NC group (Figure 6a). The expressions of miR-520h and CDKN1A in tumor tissues of xenograft models, generated by subcutaneous injection of IGF2-AS stably over-expressed HepG2 were detected by qRT-PCR assay. The results showed that miR-520h expression was decreased, and CDKN1A expression was increased after IGF2-AS over-expression (Figure 6b). Moreover, immunohistochemistry assay confirmed that IGF2-AS over-expression in HepG2 cells increased CDKN1A expression (Figure 6c), while decreased Ki-67 expression in tumor tissues of xenograft models (Figure 6d).

Taken together, our findings revealed that IGF2-AS was a tumor-suppressor, and inhibited tumorigenesis and aggressive behaviors of HCC cells via sponging miR-520h to up-regulate CDKN1A both *in vitro* and *in vivo*.

## Discussion

Although with great advances of clinical strategies, the present treatment for HCC, including surgical-, chemo-, and radiation-therapy, cannot avoid postoperative relapse, remote metastasis or poor survival due to the invasive and metastatic characteristics of HCC [18]. Therefore, molecular mechanisms of HCC progression are in urgent need to be investigated for developing novel and effective therapeutic strategies. In current study, we first revealed that IGF2-AS was significantly down-regulated in the HCC patient tissues than in the normal control tissues based on the high-throughput RNA-seq data from both the TCGA database and the GEPIA database. The widespread application of high-throughput technology allows the rapid and precise genotyping of large number of biomarkers; however, high-throughput technologies commonly produce a substantial amount of false positive and untrue results due to the data quality issues, and the accepted statistical analyses on controlling the false positive rate and limiting the false negative rate, the expression values of the identified potential biomarkers may represent the true biological findings need to be validated by laboratory experiments [19–21]. To avoid the possible false positive results from the high- throughput technology, we collected 106 paired HCC tissues/adjacent normal tissues from the local HCC patients, and applied human cell lines (3 from HCC and 1 from normal liver) for qRT-PCR assay to verify the express profile of IGF2-AS obtained by above bioinformatics assay.

Considering IGF2-AS was not the only down-regulated lncRNA in HCC tissues, whether IGF2-AS had biological function in HCC was particular important. Herein, the prognostic value of IGF2-AS expression in HCC patient clinical outcome were further explored based on both TCGA database and local patient information. The results disclosed the direct association between decreased IGF2-AS expression with poor overall survival/disease free survival in HCC patients. More importantly, we demonstrated that IGF2-AS over-expression significantly inhibited the viability, colony formation, invasion and migration, while promoted apoptosis of HCC cells *in vitro*, and inhibited HCC xenograft growth *in vivo*. Collectively, these results indicated that IGF2-AS may serve as a tumor suppressor in HCC, a potential prognostic biomarker and therapeutic target for HCC patients. Our findings are in line with what have been reported in several other cancers including breast cancer [6], gastric cancer [7], CRC [8], and gastric adenocarcinoma [22].

The miRNAs are small endogenous non-coding RNAs that play essential roles in many biological processes, which are comprehensively dysregulated in cancer cells, contributing to cancer development via many different mechanisms, such as HCC [23,24] Increasing evidences have shown that lncRNAs may serve as ceRNAs of miRNAs to protect the target mRNAs from degradation [25–27]. IGF2-AS has been reported to play anti-cancer efficacy via ceRNA mechanism in some other cancers, such as in gastric cancer via miR-195/CREB1 axis [7] and in gastric adenocarcinoma via miR-503/SHOX2 axis [22]. These information implied that IGF2-AS may prevent the carcinogenesis of HCC as ceRNA for specific miRNAs. As expected, our further studies confirmed that IGF2-AS could competitively sponge miR-520h in HCC cells. Dysregulated miR-520h has been reported be involved in cancer development and drug-resistance. Studies have reported that downregulation of miR-520h contributes to anticancer activity in human breast cancer and human cervical cancer cell lines [28]; miR-520h promotes drug resistance to paclitaxel by targeting OTUD3-PTEN axis in breast cancer [29]; miR-520h overcomes bortezomib resistance in multiple myeloma via suppressing APE1 [30]. However, the functions of miR-520h in carcinogenesis and metastasis of HCC have not been reported. In line with the literatures, in current study we found that miR-520h was significantly up-regulated in liver cancer tissues based on TCGA database, HCC tissues from local patients and HCC cells.

Evidences have indicated that miRNAs can directly interact with the 3ʹ-UTRs of mRNAs, and inhibit the mRNA translation [31]. Subsequently, we investigated miRNA sponged by IGF2-AS and target mRNA of miRNA. Bioinformatics assay found that miR-520h had potential binding sites in the 3′-UTR sequences of IGF2-AS and CDKN1A. CDKN1A (p21) is a key cell-cycle inhibitor, and an effector of TP53, presenting an essential role in HCC progression [32]. Knowledge of the regulation and function of CDKN1A in cancer cells has opened up several areas of investigation and led to novel therapeutic strategies. Some miRNAs have been identified to be associated with CDKN1A expression [33–35]. The direct binding between miR-520h with CDKN1A was verified by dual-luciferase activity. Furthermore, the expression of IGF2-AS was negatively associated with miR-520h expression and a significant reciprocal repression feedback loop present in HCC cells, while IGF2-AS expression was positively correlated with CDKN1A in HCC cells. Moreover, miR-520h over-expression or CDKN1A silencing reversed IGF2-AS inhibited aggressive behaviors of HCC cells. As a result, this work provided new evidences supporting that IGF2-AS served as a ceRNA of miR-520h to up-regulate CDKN1A expression. Since the importance of CDKN1A in the cell cycle and HCC development, our findings revealed a therapeutic implication of IGF2-AS/miR-520h/CDKN1A pathway in HCC patients.

## Conclusion

Our current work revealed a down-regulated IGF2-AS in HCC cells and tissues, which was significantly associated with poor prognosis of HCC patients. Up-regulated IGF2-AS inhibited HCC growth and metastasis by sponging miR-520h to up-regulate CDKN1A expression. Therefore, targeting IGF2-AS/miR-520h/CDKN1A axis would provide new biomarkers for early diagnosis, therapeutics and prognosis in HCC patients. Future studies on the biological function and mechanism of IGF2-AS/miR-520h/CDKN1A axis in HCC carcinogenesis and distal organ metastasis should be performed *in vivo*, using the HCC orthotopic xenograft model mice with distal organ metastasis, before the clinical translational research was carried out.

## Data Availability

The datasets used and/or analyzed during the current study are available from the corresponding author on reasonable request.

## Highlights

1. LncRNA IGF2-AS is a tumor-suppressor in HCC.
2. Lower IGF2-AS expression is associated with poor prognosis of HCC patients.
3. LncRNA IGF2-AS inhibits HCC oncogenesis and development by IGF2-AS/miR-520h/CDKN1A pathway.
4. LncRNA IGF2-AS may serve as a new biomarker for HCC management.
